# Ontogenic Development of Th1 and Th2 Cytokine Capabilities in Random Bred Mice

**DOI:** 10.1080/10446670290030963

**Published:** 2002-03

**Authors:** Omar R. Fagoaga, Sandra L. Nehlsen-Cannarella

**Affiliations:** Department of Pathology, Immunology Center Loma Linda University School of Medicine and Medical Center 11234 Anderson Street Loma Linda CA 92354-2870 USA

## Abstract

Neonatal mouse Th1 capabilities mature by postnatal day 5. Neonatal T cells have been reported to exhibit a bias towards Th2 cytokine production when co-cultured with adult antigen presenting cells (APC). We studied mouse T cells co-cultured with contemporary APC to evaluate neonatal cytokine production capabilities. In response to allogeneic stimulation, T cells co-cultured with contemporary APC from day 5 pups produced 37-fold greater IFNγ and 1.4-fold greater IL-2 levels than day 20 weanling mice. After CD3 ligation, cells from day 5 pups produced 4- (IL-2) and 10-fold (IFNγ) greater levels than adults (day 45), and concentrations were 27- (IL-2) and 18-fold (IFNγ) higher than with allogeneic stimulation alone. On average, the percent difference in concentrations was 418 (IL-4), 286 (IL-2) and 1140% (IFNγ) higher in unseparated spleen cells than in isolated splenic CD4 cells and APC. These results demonstrate that, in response to allogeneic stimulation with or without CD3 ligation, lymphocytes of neonatal mice (day 5) have the capacity to produce equivalent or greater TcR-dependent Th1 cytokine (IL-2 and IFNγ) levels than adult mice. Findings also support the idea that the reported Th2 bias of neonatal T cells may be the result of in vitro manipulation and choice of mouse strain, not of an inherent bias.


*Developmental Immunology*, 2002 Vol. 9 (1), pp. 1–8

Taylor & Francis healthsciences

### Ontogenic Development of Th1 and Th2 Cytokine Capabilities in Random Bred Mice

OMAR R. FAGOAGA* and SANDRA L. NEHLSEN-CANNARELLA

*Department of Pathology, Immunology Center, Loma Linda University, School of Medicine and Medical Center, 11234 Anderson Street, Loma Linda, CA 92354-2870, USA*

*(Submitted February 2000; Accepted 19 May 2000)*

Neonatal mouse Th1 capabilities mature by postnatal day 5. Neonatal T cells have been reported to exhibit a bias towards Th2 cytokine production when co-cultured with adult antigen presenting cells (APC). We studied mouse T cells co-cultured with contemporary APC to evaluate neonatal cytokine production capabilities. In response to allogeneic stimulation, T cells co-cultured with contemporary APC from day 5 pups produced 37-fold greater IFN$\gamma$ and 1.4-fold greater IL-2 levels than day 20 weanling mice. After CD3 ligation, cells from day 5 pups produced 4- (IL-2) and 10-fold (IFN$\gamma$) greater levels than adults (day 45), and concentrations were 27- (IL-2) and 18-fold (IFN$\gamma$) higher than with allogeneic stimulation alone. On average, the percent difference in concentrations was 418 (IL-4), 286 (IL-2) and 1140% (IFN$\gamma$) higher in unseparated spleen cells than in isolated splenic CD4 cells and APC. These results demonstrate that, in response to allogeneic stimulation with or without CD3 ligation, lymphocytes of neonatal mice (day 5) have the capacity to produce equivalent or greater TcR-dependent Th1 cytokine (IL-2 and IFN$\gamma$) levels than adult mice. Findings also support the idea that the reported Th2 bias of neonatal T cells may be the result of *in vitro* manipulation and choice of mouse strain, not of an inherent bias.

*Keywords*: Neonate; Development; Mouse; IL-2; IFN$\gamma$; IL-4

### INTRODUCTION

In previous experiments, critical immune cell types were identified and enumerated between birth and maturity (Fagoaga *et al.*, 2000). While peripheralization of B, NK and T lymphocyte subsets is requisite for immune maturation, population of lymphoid compartments is only part of the formula for survival. Mature functional capabilities are also required, and particularly critical is the production of cytokines. Mosmann *et al.* (1986) have categorized T helper cells based on the cytokine profile they produce, those that promote cell-mediated or inflammatory immunity (Th1 = IL-2, IFN$\gamma$) versus those that promote antibody-mediated humoral immunity (Th2 = IL-4 and IL-10). These distinct pathways of T helper cell differentiation are functionally exclusive of each other and act to regulate immune responses (Oppenheim, 1990). Adkins *et al.* (1993) has reported that the immune status of 4-day-old neonatal BALB/c inbred mice is Th2 dominant, a characteristic that predominates throughout the neonatal period, and that adult-like Th1 production is achieved only by postnatal day 45. In their study, activation of lymph node T cells from infant mice by CD3 ligation in the presence of adult syngeneic antigen presenting cells produces abundant IL-4 (Th2 cytokine) but little IL-2 (a Th1 cytokine). Th1 production was established by 5–6 weeks of age and high IL-4 production during development was thought to contribute to the inherent inability of young helper cells to produce Th1 cytokines.

In defining the functional (cytokine) profile of neonatal cells, Adkins *et al.* (1993) co-cultured CD4+ cells with syngeneic adult antigen presenting cells (APC:Th $<$ 2:1 ratio). This experimental paradigm was designed to clarify mechanisms underlying observed immaturity of neonatal T cells. Adult rather than contemporary APC were used as costimulators, a decision based on reports of neonatal APC immaturity. Although, some investigators contend

*Corresponding author. Tel.: +1-313-966-0936. E-mail: ofagoaga@dmc.org

ISSN 1044-6672 print © 2002 Taylor & Francis Ltd
DOI: 10.1080/10446670290030963

that neonatal APC function is immature (Hunt *et al.*, 1994), others report a 100-fold increase in efficiency over those of adults (Van Tol *et al.*, 1983; 1984a,b; Marwitz *et al.*, 1988). Since lymphocytes and APC have evolved to function cooperatively, it is logical that function of one cell type at a specific age would be coordinated with that of the other cell type at the same age. With this rationale, neonatal lymphocytes would be expected to function optimally when cooperating with APC of the same stage of development. In the mouse, there has been no study where contemporary APC have been used to test neonatal cytokine production (for review see Adkins, 1999). Therefore, in the following experiments, and unique to this work, functional maturation of neonatal helper lymphocytes was evaluated in context of contemporary rather than adult accessory cells. Also novel to these culture experiments was the preservation of age-specific Th-to-APC ratios by culturing uncorrupted mixed-cell preparations. This decision was a logical extension of our immunophenotyping data (Fagoaga *et al.*, 2001) illuminating dynamic changes occurring in neonatal spleen cell populations. Without consideration of cell proportions, erroneous assessment of functional capabilities by sorted, isolated cells in culture is probable. Although, an alternative approach would have been to assay for optimal responses from various ratios of Th-to-APC in culture, optimal *in vitro* responses do not necessarily reproduce *in vivo* conditions reflecting age-appropriate cell ratios and accessory function. Using natural mixed-cell cultures, we have demonstrated that newborn mice possess the capacity to produce adult-like Th1 cytokine levels in response to allogeneic stimulation and ligation of CD3 surface molecules.

### RESULTS

### Developmental Profile of Cytokine Production by Single Cell Suspensions of Spleens (SCSS) in Response to 24 h of Allogeneic Stimulation (Mixed Lymphocyte Cultures Between Members of Single Outbred Colony)

Significant age-related changes in Th1 but no Th2 cytokine production were observed after 24 h of allogeneic stimulation without CD3 ligation (Figs. 1 and 2; ANOVA, $p < 0.05$). Highest IL-2 levels were produced by cells from 5- and 10-day-old pups and day 10 production was 4-fold greater than 3- and 4-day-old pups (Fig. 1). In contrast, IFNγ production on day 5 was as much as 7-fold greater than younger pups (Fig. 2). Cells from 10- and 20-day-old pups qualitatively had a greater capacity to produce IL-2 than IFNγ. In contrast after 24 h of allogeneic stimulation, IL-4 could not be detected in cultures of cells from any age. Thus, it was demonstrated here that lymphocytes of neonatal mice (day 5) have the capacity to produce greater TcR-dependent (physiologically relevant stimuli) Th1 cytokines in response to allostimulation relative to older pups.

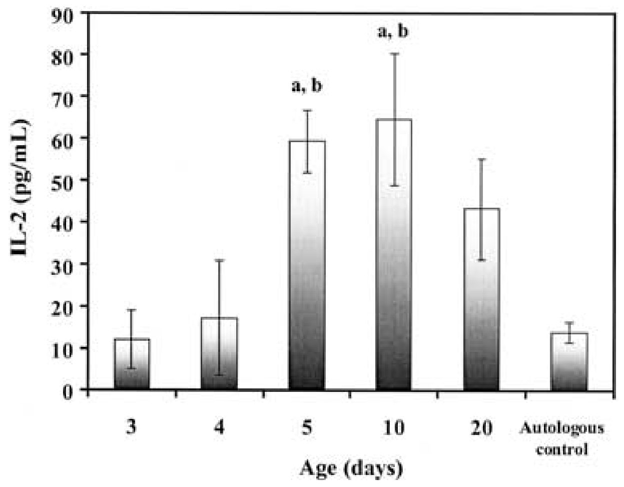

FIGURE 1 Developmental profile of IL-2 production by spleen cells of CD-1 outbred mice in response to allogeneic stimulation in 24-h mixed lymphocyte cultures. Each culture was an aliquot containing $1 \times 10^6$ CD3CD4 splenocytes. Control cells were incubated in complete media only. Data are expressed as mean ± SE of 3 samples for days 3–10, and 9 samples for day 20. Samples were comprised of pools of varying size of randomized pups as follows: days 3–10, 10 pups/pool; day 20, 2–3 pups/pool. One, day 45 mouse provided cells for autologous cultures. Statistical significance ($p < 0.05$; $df = 5$, $F = 3.86$) among ages was derived from one-way ANOVA with a Duncan's multiple range test, and is indicated by "a" versus day 3 and "b" versus day 4.

### Developmental Profile of Cytokine Production by Single Cell Suspensions of Spleen (SCSS) Cells (Allogeneic Mixed Lymphocyte Cultures) Stimulated by Ligation of CD3

Polyclonal activation via CD3 ligation and allogeneic stimulation (days 3–20) induced adult-like Th1 cytokine production in pre-weanling mice. As found after allogeneic stimulation alone, both IL-2 (Fig. 3) and

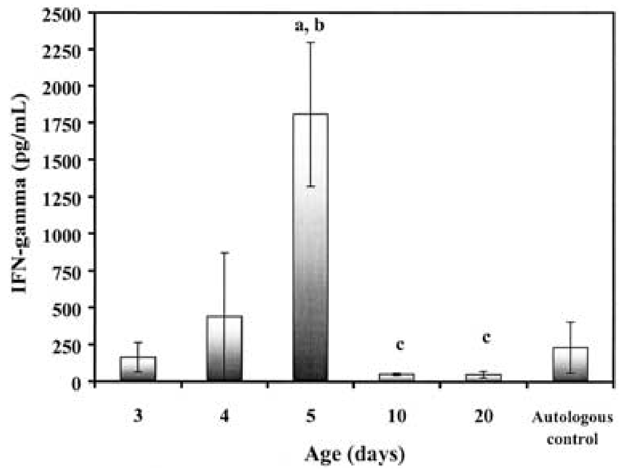

FIGURE 2 Developmental profile of IFNγ production by spleen cells of CD-1 outbred mice in response to allogeneic stimulation in 24-h mixed lymphocyte cultures. Control cells were incubated in complete media only. Data are expressed as mean ± SE of 3 samples for days 3–10, and 9 samples for days 20 and 45. Pools were as described in Fig. 1. Statistical significance ($p < 0.05$; $df = 5$, $F = 7.86$) among ages was derived from one-way ANOVA with a Duncan's multiple range test, and is indicated by "a" versus day 3; "b" versus day 4; and "c" versus day 5.

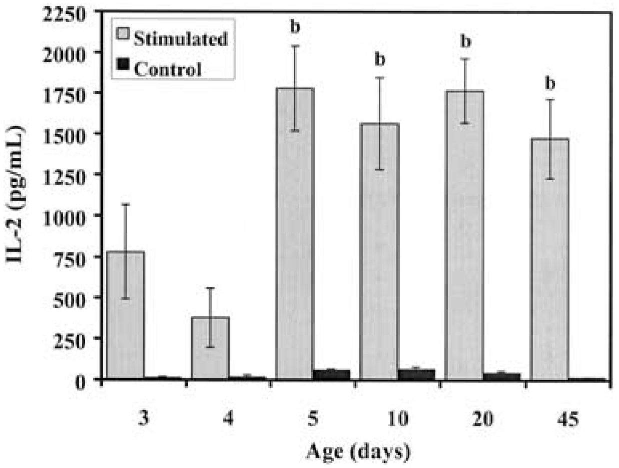

FIGURE 3 Developmental profile of IL-2 production by spleen cells of CD-1 outbred mice in response to allogeneic and polyclonal (CD3 ligation) stimulation in 24-h cultures. Each culture was an aliquot containing $1 \times 10^6$ CD3CD4 splenocytes. Control cells were incubated in complete media only. Data are expressed as mean ± SE of 3 samples for days 3–10, and 10 and 11 samples for days 20 and 45, respectively. Pools were described in Fig. 1. Statistical significance ($p < 0.05$; $df = 5$, $F = 2.85$) among ages, for experimental (not control) data, was derived from one-way ANOVA with a Duncan's multiple range test, and is indicated by "b" versus day 4.

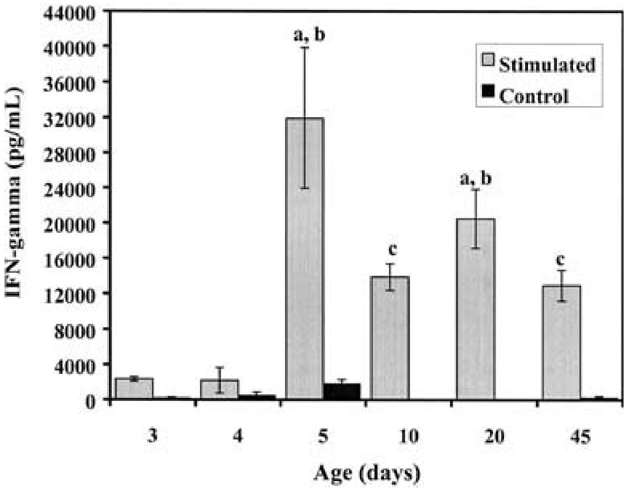

FIGURE 4 Developmental profile of IFNγ production by spleen cells of CD-1 outbred mice in response to allogeneic and polyclonal (CD3 ligation) stimulation in 24-h cultures. Each culture was an aliquot containing $1 \times 10^6$ CD3CD4 splenocytes. Control wells were incubated in complete media only. Data are expressed as mean ± SE of 3 samples for days 3–10, and 10 and 11 samples for days 20 and 45, respectively. Pools were as described in Fig. 1. Statistical significance ($p < 0.05$; $df = 5$, $F = 6.74$) among ages, for experimental (not control) data, was derived from one-way ANOVA with a Duncan's multiple range test, and is indicated by "a" versus day 3; "b" versus day 4; and "c" versus day 5.

IFNγ (Fig. 4) production varied significantly (ANOVA, $p < 0.05$) between ages. Also, IL-2 and IFNγ concentrations increased significantly by day 5. At day 5, IL-2 and IFNγ levels were on the average 4- and 10-fold higher, respectively, than levels of cells from younger pups. Remarkably, IL-2 and IFNγ concentrations produced by cells from 5-day-old mice were equivalent to and higher, respectively, than cytokine concentrations produced by cells from adult mice. Furthermore, at peak levels, the IL-2 and IFNγ concentrations induced by CD3 ligation were 27 and 18 times greater, respectively, than that induced by allogeneic stimulation alone. Moreover, relative to the plateau observed for IL-2 production beyond day 5, IFNγ concentrations decreased 2-fold by postnatal day 10; a profile also observed after allogeneic stimulation alone. In sharp contrast, Th2 cytokine production (IL-4) did not differ with age and averaged 82.5 pg/ml (SE = 8.8) (data not shown). Thus, it has been shown that, given an appropriate environment, neonatal mouse T cells are inherently capable of producing equivalent or greater quantities of Th1 cytokines than those of adults. Findings support the idea that the previously reported bias of neonatal T cells to selectively produce Th2 cytokines may be the result of an artificially contrived experimental paradigm.

### Cytokine Production by Neonatal T Helper Cells Receiving Either One or Two Signals

Th1 cytokine production by Th cells alone (sorted CD4 cells; "background" activity due to allostimulation within pools and CD3 ligation but no APC) was very low (IL-2, $< 15$ pg/ml; IFNγ, $< 100$ pg/ml on the average for 20- and 45-day-old pups) and did not change with age. IL-2 and IFNγ concentrations were 300-fold and $> 40$-fold lower, respectively, in cultures containing only Th cells than in cultures of Th cells with APC (data not shown). IL-4 concentrations were nearly undetectable from Th alone, and $< 25$ pg/ml in Th + APC. Thus, it was established that neonatal Th cells require both signals to be activated, and that these signals could be delivered by contemporary APC.

### Developmental Profile of Cytokine Production by Sorted CD4 Cells Stimulated by Allogeneic APC and CD3 Ligation

Cytokine production by isolated neonatal Th cells co-cultured with isolated contemporary APC and stimulated by CD3 ligation matured within the first week of life. Significant (one-way ANOVA, $p < 0.05$) age-related changes in IL-2 and IL-4 production, but not IFNγ, were observed. IL-2 (Fig. 5) and IFNγ (data not shown) production had reached 100% adult levels by day 3 (days 0–2 not tested). IL-2 levels crested on day 10 while IFNγ remained about the same (average, 1893 ± 196 pg/ml) throughout all time points evaluated. On the other hand, IL-4 cytokine production was within adult (day 45) range on days 3–4 (Fig. 6), and 50% of adult values on days 5, 10 and 20. These results contrast sharply to those of others in that adult-like Th1 cytokine production capacity (T cells derived from the spleen) is established in CD-1 outbred mice at a very young age ($\leq 3$ days old), but not until adulthood (5–6 weeks as in T cells derived from lymph nodes) in BALB/c inbred mice (Adkins *et al.*, 1993).

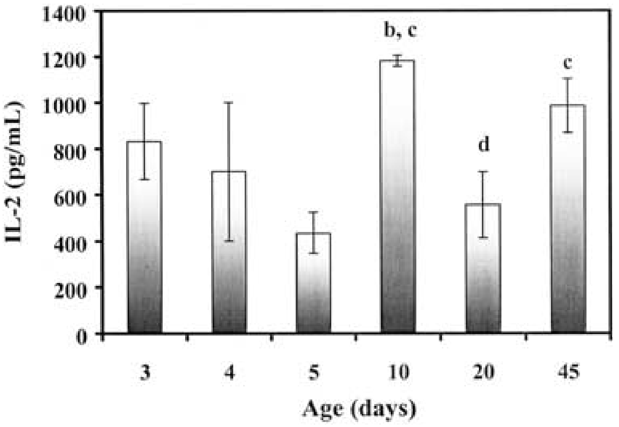

FIGURE 5 Developmental profile of IL-2 production by sorted spleen cells from CD-1 outbred mice. Each culture contained $1 \times 10^6$ sorted CD3CD4 splenocytes, $2 \times 10^6$ sorted APC, and hamster anti-mouse CD3 monoclonal antibody. Data are mean ± SE of $n = 6$, day 3; $n = 4$, day 4; $n = 9$, day 5; $n = 7$, day 10; $n = 10$, day 20; and $n = 11$, day 45. Pools were as described in Fig. 1. Statistical significance ($p < 0.05$; $df = 5$, $F = 4.73$) among ages was derived from one-way ANOVA with a Duncan's multiple range test, and is indicated by "b" versus day 4; "c" versus day 5; and "d", versus day 10.

CD3 ligation-induced cytokine production by uncorrupted SCSS was higher than the cytokine production in cultures of sorted CD4 cells plus sorted APC. Isolated (sorted) Th and APC co-cultures did not function optimally, in spite of polyclonal (CD3 ligation) stimulation and culturing at consistent ratios (<2) of APC-to-Th cells at all ages evaluated. On the average, the percent difference in cytokine concentrations was 418 (IL-4), 286 (IL-2) and 1140% (IFNγ) higher in uncorrupted SCSS cell preparations than in isolated spleen cells (sorted CD4 and APC) (Fig. 7). Production of both Th1 and Th2 cytokines was greater in uncorrupted spleen cells than in purified Th-APC cultures (two-way ANOVA, $p < 0.05$) for most time points evaluated. The IL-2, IFNγ and IL-4 concentrations were different between these two types of cultures ($t$-test, $p < 0.05$) on postnatal days 5 and 20, except IFNγ, which also differed on day 10. Of the cytokines studied, IFNγ was most affected by the absence of some critical component(s) in the isolated cell cultures. Therefore, composition of cell mixtures significantly affects cytokine production (age-related differences and magnitude of production) and therefore impacts interpretation of immune cell capabilities.

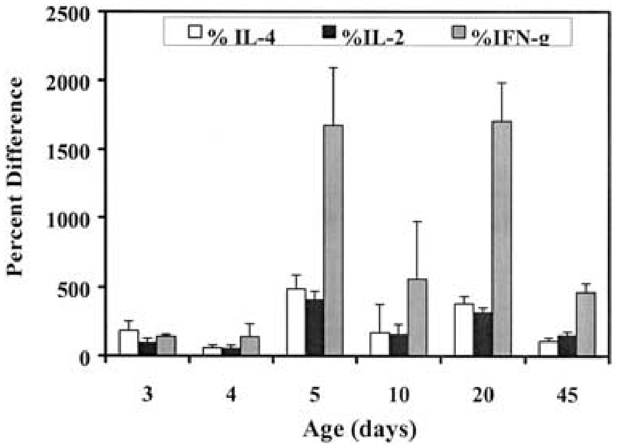

FIGURE 7 Percent increase in cytokine production of single cell suspensions of spleens compared to that of sorted Th and sorted APC from spleens of CD-1 outbred mice (male and female) at various ages. Data are expressed as mean percent increase in cytokine production by unsorted spleen cell cultures over that produced by sorted Th/APC cultures.

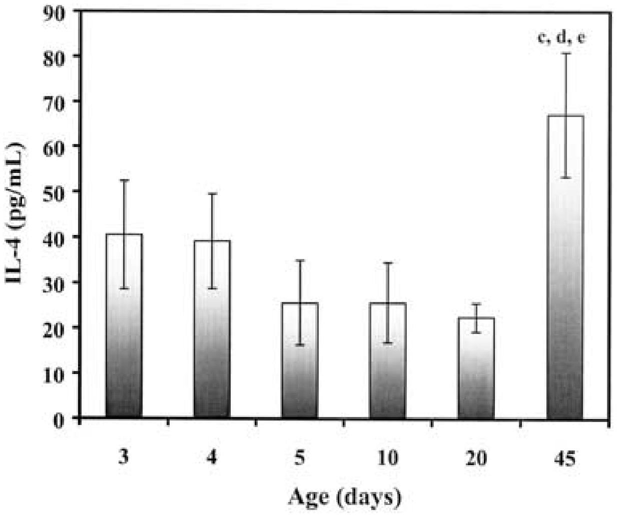

FIGURE 6 Developmental profile of IL-4 production by sorted spleen cells from CD-1 outbred mice. Each culture contained $1 \times 10^6$ sorted CD3CD4 splenocytes, $2 \times 10^6$ sorted APC, and hamster anti-mouse CD3 monoclonal antibody. Experiment and control cultures, numbers of cultures, and pools are as described in Fig. 5. Statistical significance ($p < 0.05$) among ages was derived from one-way ANOVA with a Duncan's multiple range test, and is indicated by "c", versus day 5; "d", versus day 10, and "e", versus day 20. For this data set, degrees of freedom were 5 with an F statistic of 3.19.

### DISCUSSION

These experiments with random bred mice demonstrate that Th1 capability is achieved within the first week of life. By day 5, splenocytes produced adult-like or greater IL-2 and IFNγ levels in response to allogeneic stimulation *in vitro*. The relatively modest production of IFNγ at 5 days was nearly twice that of adults, about six times higher than those reported by others (Adkins *et al.*, 1993). This variance from previous reports may be attributable to differences in cell culture composition (uncorrupted cell population ratios versus contrived ratios of two isolated cell populations), different (contemporary versus non-contemporary) APC sources, and different mouse strains (CD-1 versus BALB/c). Yet Adkins *et al.* (1993) reported that cells of 4-day-old mice produced 400% of adult IL-4 levels, a response that they found had diminished to <200% in mice only 1-day older. Given the antagonistic effects of Th1 and Th2 cytokines (Mosmann *et al.*, 1986), the results of both studies can be explained. However, the low level of Th1 cytokine production induced by allogeneic stimulation alone may be due to any one of several reasons. Indirect recognition of alloantigens (antigen processing and recognition; Liu *et al.*, 1993), cannot be achieved in 24 h, and direct recognition by naïve cells of young individuals is likely to be less than optimal. Pools containing multiple members of disparate individuals had few cells of each clone that could respond to alloantigen and produce cytokine. Hence, to further demonstrate whether neonatal cells are capable of directing mature Th1 reactions, T cells were activated through polyclonal stimulation, ligation of CD3 cell membrane molecules with hamster anti-mouse CD3 monoclonal antibody as (Adkins *et al.*, 1993) in addition to alloantigen exposure (mixed lymphocyte spleen cell cultures).

Cytokine production capability at day 5 was yet more evident when cells were maximally stimulated (polyclonal) through CD3 ligation in the presence of allogeneic antigens. Wakil *et al.* (1998) have shown that IFNγ, required to maintain IL-12 responsiveness by T cells, is requisite for establishing Th1 activity. In this study, IFNγ production by T cells of 5-day-old neonates clearly indicates that the capacity for Th1 responses is present in the immune cells of young neonates. However, high production of Th1 at this young age was solicited by extraordinary means, indicating the presence of other immunoregulatory controls perhaps safeguarding against inappropriate aggressive activity during development.

The sudden reduction in IFNγ production after day 5 is not clear. Perhaps abundant production by younger pups reflects endogenous priming of T cells undergoing peripheral self-education. Self-memory differentiation of Th1 cells is known to require IFNγ (Alferink *et al.*, 1998) and may be complete in mice by day 5. Alternatively, naïve CD4 cells exit the thymus to begin populating the spleen by day 3. The filling of splenic compartments with naïve CD4 cells reaches a peak by day 10 with a subsequent two-fold decrease by day 20 (Fagoaga *et al.*, 2000). Between days 3 and 5 the thymic output of naïve T cells is greater than at any other time. These naïve cells are the first cells to encounter self-antigen that modulates their function to produce high levels of IFNγ (Goldrath *et al.*, 2000). These changes in immunophenotype proportions and cytokine production capabilities may explain the differences in IFNγ production during this period of development.

These findings do not support the reported inability of neonatal cells to produce Th1 cytokines. Previously, Adkins *et al.* (1993) reported that neonatal T cells are intrinsically unable to produce IL-2 and IFNγ until day 45, a finding in conflict with data of the present study. At least two explanations of this discordance can resolve the discrepancy between Adkins' and our findings. First, Adkins and Hamilton (1992) and Adkins *et al.* (1993) used APC from adult mice to co-stimulate neonatal T cells in the presence of CD3 ligation. Secondly, they used an APC:T cell ratio lower than normally found in neonates. Contemporary APC used in that same ratio (<2:1) solicited significant Th1 cytokine production in our work. Thirdly, Adkins and Hamilton (1992) and Adkins *et al.* (1993) studied cytokine response capabilities of neonatal BALB/c mice. This mouse strain is biased towards production of IL-4 (a Th1 antagonist) and expresses inappropriate Th1 responses (Reiner and Locksley, 1995), designating this model as less than ideal for studies of maturation of Th1 responsiveness.

Further, confounding an understanding of neonatal cytokine production are the artifacts imposed by *in vitro* investigation. APC function has been reported to be immature in neonates (Clerici *et al.*, 1993; Hunt *et al.*, 1994; Trivedi *et al.*, 1997). Yet others have reported an increased efficiency in neonatal APC function over those of adults (Van Tol *et al.*, 1983; 1984a,b; Marwitz *et al.*, 1988). This inconsistency in reports of neonatal APC function caused us to approach this investigation from two directions. First, function of T cells was studied in context of relatively uncorrupted splenocyte preparations, thus preserving natural APC-to-T cell ratios as well as retaining other cell populations such as NKT cells. Secondly, in sorted-cell experiments, Th cells were co-cultured with contemporary APC. Although age-related changes in cytokine production were observed in both kinds of cultures, the concentrations of cytokine produced were 4- (IL-2 and IL-4) and 17-fold higher (IFNγ) in unsorted SCSS than sorted splenocyte cultures. In SCSS cultures, greatest concentrations of IL-2 and IFNγ were produced by 5-day-old pups, whereas, optimal production in sorted cell cultures was not achieved until pups were 10 days of age. Thus, composition and ratio of cooperating cell populations are critical ingredients in achieving natural regulatory immune processes, and are components shown in this study to be essential to faithfully eliciting age-appropriate immune responses.

In the sorted cell cultures the APCs were at less than a 2:1 ratio (APC to CD4 cell ratio) with CD4 cells, mainly due to granulocytes, which do not exhibit APC function. In contrast, in the total unsorted cell cultures this ratio could be calculated for B cells to CD3 cells only. The B:T cell ratios were: 11:1, 8:1, 4:1, 11:1, 4:1, 2:1 for 3-, 4-, 5-, 10-, 20-, and 45-day old pups, respectively (data obtained in a parallel immunophenotyping study; Fagoaga *et al.*, 2001). Indeed, APC:T cell ratios are higher when monocytes and dendritic cells are taken into account. The optimal B:T cell ratio appears to be at least 3.5:1 for day 5, which resulted in stimulating the highest concentrations of cytokine production. These findings are consistent with Ridge *et al.* (1996) who suggested the APC-to-virgin T cell ratio determines which type of immune response is produced by neonatal T cells. The significantly greater cytokine production realized by uncorrupted cell cultures is likely a result of providing an optimal environment of cell ratios and availability of cells other than APC and T helper cells such as dendritic, T cytotoxic/suppressors, NKT and NK cells, all of which contribute to the stimulatory and inhibitory effects of the cytokine *milieu*.

The present data are also consistent with reports (Delespesse *et al.*, 1998) that cytokine *milieu* together with accessory cell function (strong or weak CD28

costimulation) determine whether T cells mature to Th1 or Th2 effectors at any age. In more recent work, Adkins *et al.* (1994) have attributed the inability of neonatal T cells to achieve adult-like Th1 cytokine production to inefficiency of accessory cell stimulation. In their more recent study, double ligation (CD3 and CD28) of neonatal T cells to deliver classical activation of aggressive function (signals $1 + 2$) achieved induction of adult cytokine production in neonates equivalent to that induced by contemporary APC in our study. Furthermore, in a recent review, Adkins (1999) emphasizes the importance of neonatal APC in Th1/Th2 development and that neonatal APC may function more efficiently when confronted by a replicating than a non-replicating antigen. In our experiment, the use of contemporary APC in cultures of CD-1 mouse cells resulted in equivalent or greater than adult Th1 production by 5-day-old mice. Use of cell ratios reflecting a contemporaneous environment established that neonatal cells competently produce Th1 cytokines. Optimal production of inflammatory cytokines by day 5 affirm that neonatal splenic APC are efficient collaborators of neonatal T cells, an illustration of parallelism in ontogenic evolution.

Patterns of Th1 cytokine production are a reflection of available phenotypes. Before day 5, there are very few memory cells in spleen, while adult spleens contain many-fold greater numbers (Fagoaga *et al.*, 2001). Memory cells migrate to spleen after down-regulating CD62L expression (Forsthuber *et al.*, 1996), and have increased capacity to produce Th1 and Th2 cytokines compared to naïve cells (Bradley *et al.*, 1992). In certain conditions in young mice, naïve Th cells produce more IL-2 than memory Th cells (Kurashima and Utsuyama, 1997). In neonates, memory cells are generated in response to foreign and self (peripheral) antigens (post-thymic education; Alferink *et al.*, 1998). In a previous study (Fagoaga *et al.*, 2001), high thymic output of naïve T cells was observed between days 3 and 10. Thus, these early thymic naïve emigrants may be involved in self-antigen recognition and tolerization, an activity that would induce changes in cytokine responses. Cells found in spleen of day 5 pups may be the first thymic emigrants having processed peripheral tissue antigens (peripheral tolerization), at which time they "masquerade" as memory cells, perhaps later reverting to naïve T cells (Goldrath *et al.*, 2000).

In summary, the results of these culture experiments have established that neonatal outbred mice possess the capacity to produce adult-like Th1 cytokines. As reviewed for adult cells, the immune system of neonates is a complex set of cellular and soluble components that is an integral part of a larger system (neuro-endocrine-immune). If immune components are to be examined *ex vivo*, it may be important to maintain (or faithfully recreate) as much of the natural *milieu* as possible to obtain data relevant to normal function. Information generated from isolated immune components can be readily misinterpreted, a principle validated when comparing results of uncorrupted versus contrived mixtures of cells in these culture experiments. Providing different environments (spleen cell suspensions being more similar versus sorted purified Th/APC preparations being different) than those found *in vivo* yielded entirely different results within the same animal model. Findings in the experiments of Adkins *et al.* (1993) were confounded by the mouse strain selected for defining cytokine production capabilities. The BALB/c inbred mouse strain is recognized for being biased to produce strong Th2 reactions and inappropriate Th1 responses due to an inborn failure to down-modulate IL-4 production (Reiner and Locksley, 1995). In fact, it could be argued that any inbred strain might express dysregulation of one kind or another by virtue of being inbred.

### MATERIALS AND METHODS

### Animals

Timed-pregnant CD-1 mice, a random bred strain, were purchased from Charles River Laboratories (Wilmington, MA) from which the progeny were used for this study. The progeny were weaned at postnatal day 20 and housed in groups of 3–5/cage with microfilters and wood bedding. Cages were maintained in microflow ventilated racks (Allentown Caging equipment, NJ) within one room on a 12- and 12-h light–dark cycle. The pregnant animals were fed high protein diet, Harlan Teklad 7004/S-2335, (Harlan Teklad, Wisconsin) and progeny were weaned onto standard rodent chow.

### Collection of Blood and Spleen

From birth (day 0) to day 5, and again on day 10, mice were decapitated and spleens collected as pools comprised of randomly sorted pups from different litters. Six pools were created at each age for the younger animals (5–14 pups/pool on days 3, 4, 5 and 10; 2/pool for day 20) and individual spleens were studied from 45-day-old adults ($n = 6$). Spleens were excised (devoid of mesenteric fatty tissue), weighed and placed in RPMI 1640 medium at room temperature until lymphocyte harvest ($< 30$ min). Lymphocytes were collected by gently pressing the tissue through a fine nylon mesh sieve. Leukocytes in spleen cell suspensions were counted on a hemacytometer (Unopette, Becton Dickinson, Franklin Lakes, NJ). The 45-day-old mice are considered adult, as they are postpubertal and reproductively mature. Functionally, as reviewed by Mosier and Cohen (1975), the proliferative capacity of T cells, in response to mitogens (PHA and ConA) and alloantigens, does not mature before the third week after birth. Proliferative responses by splenic T and B cells are also not fully developed until 3–4 weeks of age. Capacity for T cell-dependent antibody production is not achieved until 6 weeks of age. Furthermore, cytokine production is not mature until 45 days of age (Adkins *et al.*, 1993).

### CD4 Cell Isolation

CD4 splenocytes were positively sorted with "DYNABEADS™ Mouse CD4" (Dynal, Lake Success, NY), immunomagnetic beads conjugated with rat anti-mouse CD4 (L3T4) monoclonal antibody. Mixtures of cells and dynabeads were rocked 45 min at 4°C to bind the antibody-bead complex to cells. Subsequently, CD4 cells were captured with a magnet held against the test tube; non-CD4 cells were collected as free cells in suspension in aspirated supernatant. Free non-CD4 cells served as antigen presenting cells (see below). Positively-sorted CD4 cells were obtained when disassociated from cell-immunobead conjugates (DETACHaBEAD Mouse CD4, Dynal, Lake Success, NY). Briefly, isolated CD4 cells ($1 \times 10^6$ cells/100 μl) were incubated with 20 μl DETACHaBEAD reagent for 45–60 min at room temperature. After beads were released from cells and collected by magnet, detached CD4 cells were collected in supernatant, washed twice with RPMI 1640, and resuspended in complete media.

### Accessory Cell Isolation ("Sorted APC")

APC were purified (enriched) by negative sorting (treating aspirated supernatant with DYNABEADS conjugated to Thy-1.2 mAb, Dynal, Lake Success, NY). This treatment depleted remaining CD8 cells and any CD4 cells not previously captured by the CD4 positive selection. The remaining cells were composed of B cells (an average of 90% CD19+ lymphocytes), monocytes and very few granulocytes (based on side and forward scatter light characteristics) as assessed by flow cytometry.

### Quantification of Percent CD4 Cells, and Purity of Sorted CD4 and APC

Two-color immunofluorescence was used to quantify percent splenic CD4 cells, and purity of CD4 and APC cell isolation experiments. Lymphocyte suspensions were stained (hamster anti-mouse CD3*FITC, Caltag Laboratories, San Francisco, CA; and rat anti-mouse CD4* or CD8*PE, PharMingen, San Diego, CA) and analyzed as previously described. The sorting experiments yielded greater than 95% purity of CD3+ CD4+ cells. The APC population contains at least two subsets of cells with antigen presenting capabilities, B cells and monocytes, and was devoid of T cells. This procedure yielded >95% Thy1.2-negative cells. Thy1.2-negative cells (APC) were washed in RPMI 1640 and resuspended in complete media.

### T Cell Stimulation Assay

Sorted CD4 co-cultured with sorted APC or uncorrupted spleen cell preparations were stimulated (polyclonal) by ligation with anti-CD3 monoclonal antibody. The cell clone 145-2C11 (PharMingen, San Diego, CA) producing anti-CD3 monoclonal antibody had been grown in T-150 flasks with complete media. 145-2C11 cells were allowed to grow to culture media exhaustion, when most cells were dead. The conditioned media was collected and centrifuged at 400*g* for 30 min at 4°C to eliminate cells and cellular debris. Optimal concentration of anti-CD3 antibody was determined to be the serial dilution (8-fold) yielding the highest T cell proliferation in 42 h as assessed by quantifying $^3$H-thymidine incorporation during an 18-h radioactive pulse. Splenocytes were collected from spleens as described above. Subsequently, cell cultures were set up in 24-well plates (Fisher Scientific, Pittsburgh, PA). Cultures consisted of either uncorrupted single cell spleen suspensions (SSCS), or mixtures of sorted CD4 splenocytes ($1 \times 10^6$/culture) and sorted APC ($2 \times 10^6$/culture) and 1 ml of optimally diluted CD3 monoclonal antibody. To obtain a constant number of CD4 cells in each culture, absolute numbers of CD4 cells in uncorrupted cell preparations were determined by flow cytometry and an aliquot of splenocytes containing $1 \times 10^6$ CD4 cells was incubated with 1 ml CD3 monoclonal antibody. Each culture volume was brought up to 2 ml with complete media and incubated at 37°C in 5% $CO_2$ in 100% humidified air. CD3 antibody was not added to control cell cultures. After 24 h, supernatants were collected and stored frozen (−70°C) until assessed for cytokines.

### Cytokine Assays

Commercial cytokine ELISA kits from ENDOGEN (Woburn, MA) were used to assess IL-2, IL-4 and IFNγ production by murine splenocytes after CD3 ligation. Assay procedures followed manufacturer's instructions. Briefly, standard sandwich ELISA utilizing capture antibody (96-well microculture plates; Fisher Scientific, Pittsburgh, PA) and enzyme-conjugated detection antibody was employed. Plates were read in a Mark II Plate Reader (Dynex Technologies Inc, Chantilly, VA) at appropriate wavelengths indicated for each cytokine kit. Throughout this study, two (one high, one low) control samples of known concentrations of specific cytokine were included in each assay to assure reproducibility. Standards and samples were assayed in duplicate and samples from all ages were run simultaneously to ensure consistent and comparable results across age groups. The assay sensitivities were 3 pg/ml for IL-2, 5 pg/ml for IL-4, and 15 pg/ml for IFNγ.

### Statistical Analysis

One-way ANOVA and Duncan's post hoc test for multiple comparisons among different ages were calculated. If test for homosedasticity was significant (greater than the critical value for F-max test for homogeneity of variances), data were log transformed. After transformation, if significant homogeneity of variances persisted, a Kruskal–Wallis ANOVA with multiple comparisons was performed to assess differences among age groups. $p < 0.05$ was considered significant.

### Acknowledgements

This work is part of the doctoral thesis research (Fagoaga, June 1999; Loma Linda University). We wish to thank Cathy Hisey, Catherine Gaffney and Judy Folz-Holbeck for technical assistance in the conduct of the study.

### References

Adkins, B. (1999) "T cell function in newborn mice and humans", *Immunology Today* **20**(7), 330–335.

Adkins, B. and Hamilton, K. (1992) "Freshly isolated, murine neonatal T cells produce IL-4 in response to anti-CD3 stimulation", *Journal of Immunology* **149**, 3448–3455.

Adkins, B., Ghanei, A. and Hamilton, K. (1993) "Developmental regulation of IL-4, IL-2 and IFN-γ production by murine peripheral T lymphocytes", *Journal of Immunology* **151**, 6617–6626.

Adkins, B., Ghanei, A. and Hamilton, K. (1994) "Up-regulation of murine neonatal T helper cell responses by accessory cell factors", *Journal of Immunology* **153**, 3378–3385.

Alferink, J., Tafuri, A., Vestweber, D., Hallmann, R., Hammerling, G.J. and Arnold, B. (1998) "Control of neonatal tolerance to tissue antigens by peripheral T cell trafficking", *Science* **282**, 1338–1341.

Bradley, L.M., Atkins, G.G. and Swain, S. (1992) "Long-term CD4 memory T cells from the spleen lack MEL-14, the lymph node homing receptor", *Journal of Immunology* **148**, 334–341.

Clerici, M., DePalma, L., Roilides, E., Baker, R. and Shearer, G.M. (1993) "Analysis of T helper and antigen-presenting cell functions in cord blood and peripheral blood leukocytes from healthy children of different ages", *Journal of Clinical Investigations* **91**, 2829–2836.

Delespesse, G., Ohshima, Y., Demure, C., Shu, U., Byun, D.G. and Sarfati, M. (1998) "Maturation of human neonatal CD4+ and CD8+ lymphocytes into Th1/Th2 effectors", *Vaccine* **16**(14–15), 1415–1419.

Fagoaga, O.R., Yellon, S.M. and Nehlsen-Cannarella, S.L. (2001) "Maturation of lymphocyte immunophenotype and memory T helper cell differentiation during development in mice", *Developmental Immunology* **8**, 47–60.

Forsthuber, T., Yip, H.C. and Lehmann, P.V. (1996) "Induction of TH1 and TH2 immunity in neonatal mice", *Science* **271**, 1728–1730.

Goldrath, A.W., Bogatzki, L.Y. and Bevan, M.J. (2000) "Naïve T cells transiently acquire a memory-like phenotype during homeostasis-driven proliferation", *The Journal of Experimental Medicine* **192**(4), 557–564.

Hunt, D.W.C., Huppertz, H.I., Jiang, H.J. and Petty, R.E. (1994) "Studies of human cord blood dendritic cells: evidence for functional immaturity", *Blood* **84**, 4333–4343.

Kurashima, C. and Utsuyama, M. (1997) "Age-related changes of cytokine production by murine helper T cell subpopulations", *Pathobiology* **65**(3), 155–162.

Liu, Z., Sun, Y.K., Xi, Y.P., Maffei, A., Reed, E., Harris, P. and Suciu-Foca, N. (1993) "Contribution of direct and indirect recognition pathways to T cell alloreactivity", *Journal of Experimental Medicine* **177**, 1643–1650.

Marwitz, P.A., van Arkel-Vigna, E., Rijkers, G.T. and Zegers, B.J.M. (1988) "Expression and modulation of cell surface determinants on human adult and neonatal monocytes", *Clinical Experimental Immunology* **72**, 260–266.

Mosier, D.E. and Cohen, P.L. (1975) "Ontogeny of mouse T-lymphocyte function", In: Paul, W.E., Session Chairman, *Development and aging in organ systems* (FASEB: Atlantic City, NJ, April, 1974) pp. 137–140.

Mosmann, T.R., Cherwinski, H., Bond, M.W., Giedlin, M.A. and Coffman, R.L. (1986) "Two types of murine helper T cell clone. I. Definition according to profiles of lymphokine activities and secreted proteins", *Journal of Immunology* **136**, 2348–2357.

Oppenheim, J.J. (1990) *Molecular and Cellular Biology of Cytokines* (Wiley, New York), pp. 204–235.

Reiner, S.L. and Locksley, R.M. (1995) "The regulation of immunity to Leishmania major", *Annual Reviews of Immunology* **13**, 151–177.

Ridge, J.P., Fuchs, E.J. and Matzinger, P. (1996) "Neonatal tolerance revisited: turning on newborn T cells with dendritic cells", *Science* **271**, 1723–1726.

Trivedi, H.N., Hay-Glass, K.T., Gangur, V., Allardice, J.G., Embree, J.E. and Plummer, F.A. (1997) "Analysis of neonatal T cells and antigen presenting functions", *Human Immunology* **57**, 69–79.

Van Tol, M.J.D., Zijlstra, J., Heijnen, C.J., Kuis, W., Zegers, B.J.M. and Ballieux, R.E. (1983) "Antigen-specific plaque-forming cell response of human cord blood lymphocytes after *in vitro* stimulation by T cell-dependent antigens", *European Journal of Immunology* **13**, 390–397.

Van Tol, M.J.D., Zijlstra, J., Zegers, B.J.M. and Ballieux, R.E. (1984a) "Antigen-specific plaque-forming cell responses in cultures of peripheral blood mononuclear cells of human neonates and infants", *Journal of Pediatrics* **105**, 738–744.

Van Tol, M.J.D., Zijlstra, J., Zegers, B.J.M. and Ballieux, R.E. (1984b) "Distinct role of neonatal and adult monocytes in the regulation of *in vitro* antigen-induced plaque-forming cell response in man", *Journal of Immunology* **134**, 1902–1908.

Wakil, A.E., Wang, Z.E., Ryan, J.C., Fowell, D.J. and Locksley, R.M. (1998) "Interferon gamma derived from CD4(+) T cells is sufficient to mediate T helper cell type 1 development", *Journal of Experimental Medicine* **188**, 1651–1656.

